# Automated Electrical Resistivity Tomography for Continuous Monitoring of Permafrost Dynamics: First Field Application and Validation in Central Asia

**DOI:** 10.3390/s26175621

**Published:** 2026-09-04

**Authors:** Mohammad Farzamian, Tamara Mathys, Christin Hilbich, Teddi Herring, Martin Hoelzle, Azamat Sharshebaev, Miguel Esteves, Erich Lippmann, Arne Schwab, Christian Hauck

**Affiliations:** 1 Instituto Nacional de Investigação Agrária e Veterinária, 2780-157 Oeiras, Portugal; 2 Centre for Geographical Studies, Associate Laboratory TERRA, IGOT, Universidade de Lisboa, 1600-276 Lisbon, Portugal; miguele@edu.ulisboa.pt; 3 Department of Geosciences, University of Fribourg, 1700 Fribourg, Switzerland; tamara.mathys@unifr.ch (T.M.); christin.hilbich@unifr.ch (C.H.); martin.hoelzle@unifr.ch (M.H.); christian.hauck@unifr.ch (C.H.); 4 Department of Civil Engineering, University of Calgary, Calgary, AB T2N 1N4, Canada; teddi.herring@ucalgary.ca; 5 Central-Asian Institute for Applied Geosciences, Bishkek 720027, Kyrgyzstan; a.sharshebaev@caiag.kg; 6 Lippmann Geophysical Instruments (LGM), 94571 Schaufling, Germany; lippmann@l-gm.de; 7 Schwab Research Technology, 28832 Achim, Germany; aschwab@schwartech.de

**Keywords:** autonomous electrical resistivity tomography (A-ERT), permafrost monitoring, freeze–thaw dynamics, long-term environmental monitoring, temperature–resistivity hysteresis, Central Tien Shan

## Abstract

Continuous monitoring of permafrost dynamics remains challenging in remote high-mountain environments due to logistical constraints, harsh climatic conditions, and the limited availability of spatially distributed observations. In addition to direct temperature observations in boreholes, Autonomous Electrical Resistivity Tomography (A-ERT) offers significant potential for long-term monitoring by providing high temporal resolution observations of subsurface electrical properties, which are highly sensitive to freeze/thaw processes. This study presents the field validation of a low-power A-ERT system designed for long-term autonomous operation in extreme environments. The system was deployed at a high-altitude permafrost site near the Kumtor gold mine in the Central Tien Shan, Kyrgyzstan, representing the first application of continuous A-ERT monitoring in the Central Asian mountain ranges. The system operated continuously under harsh environmental conditions with air temperatures as low as −30 °C. Data quality remained consistently high throughout the monitoring period, with less than 1% of measurements removed during filtering, and inversion results with root-mean-square errors generally ranging between 3% and 4%. Time-lapse resistivity observations revealed strong seasonal freeze–thaw dynamics within the active layer and continued seasonal resistivity variations within the underlying permafrost despite permanently frozen conditions. Analysis of depth-dependent resistivity–temperature relationships revealed increasingly pronounced hysteresis behavior below the active layer, indicating that subsurface electrical properties were not controlled solely by temperature. This behavior likely reflects variations in unfrozen water content and pore connectivity within the fine-grained permafrost, where liquid water can persist at sub-zero temperatures. The results demonstrate the capability of the A-ERT system for reliable long-term autonomous monitoring in remote permafrost environments and investigation of coupled thermal and hydrological processes in permafrost systems.

## 1. Introduction

Permafrost in high-mountain environments plays a key role in regulating hydrological processes, controlling slope stability, and affecting infrastructure safety, particularly under ongoing climate change. In regions such as the Tien Shan Mountains, which are known as the “water tower of Central Asia”, these processes are of increasing concern due to warming trends [1,2] and the presence of critical infrastructure such as hydropower plants [3]. However, despite increasing concern regarding permafrost degradation and its implications for hydrology, geomorphological hazards, and infrastructure stability, observational data in this region remain limited, especially when it comes to capturing both the spatial variability and temporal dynamics of the active layer and underlying permafrost [4]. Most existing monitoring efforts rely on borehole measurements [2,5,6], which provide accurate but highly localized information and are often insufficient to resolve short-term variability or lateral heterogeneity.

Geophysical methods offer a valuable complement to these traditional approaches by providing spatially distributed information on subsurface conditions. Among these, Electrical Resistivity Tomography (ERT) has been widely used to investigate permafrost, as electrical resistivity is strongly influenced by the presence of ice and liquid water [7,8,9]. Numerous studies have demonstrated the applicability of ERT for mapping permafrost extent, estimating active layer thickness, and monitoring seasonal freeze–thaw processes (e.g., [10,11,12]). In particular, time-lapse ERT has proven effective for tracking temporal changes in subsurface conditions and identifying seasonal and interannual variability (e.g., [13,14,15]) and assessing unfrozen water content dynamics [16]. In the Tien Shan, however, only a few studies have investigated permafrost using geophysical methods, notably by combining ERT and Refraction Seismic Tomography (RST) within petrophysical joint inversion schemes [4] or integrating geophysical data with numerical models to assess regional thaw depth and permafrost distribution [17].

Recent developments in instrumentation have enabled automated and quasi-continuous ERT measurements, commonly referred to as Autonomous Electrical Resistivity Tomography (A-ERT). These systems significantly improve temporal resolution and allow the observation of rapid subsurface changes that are often not captured by conventional survey-based approaches [18,19]. A-ERT systems have been successfully applied in alpine (e.g., [18]), Arctic (e.g., [20,21]), and Antarctic environments (e.g., [22]), demonstrating their capability for long-term monitoring of freeze–thaw dynamics under extreme environmental conditions. Nevertheless, autonomous long-term deployments remain relatively limited, and many studies still depend on manually repeated surveys, restricting temporal resolution and increasing logistical demands in remote environments (e.g., [23]).

Despite recent advances, several methodological and technical challenges remain in the application of ERT and A-ERT to continuous permafrost monitoring. High contact resistance under frozen or dry conditions, together with reduced current penetration in highly resistive materials, can significantly affect data quality and system performance [8,14,24]. In permafrost environments, subsurface resistivity commonly exceeds 10 kΩm and may even reach MΩm levels during winter when pore water freezes and conductive pathways become strongly reduced (e.g., [25]) or when massive ice is encountered [26,27,28]. Under such conditions, resistivity systems must be capable of operating with very small injected currents while simultaneously maintaining sufficiently large voltage input ranges to remain within instrumental limits (e.g., [29]). In addition, systems intended for long-term deployment in remote mountain or polar environments must be robust, weather-resistant, low-power, and capable of autonomous operation with minimal field intervention.

Farzamian et al. [30] recently demonstrated the feasibility of long-term A-ERT monitoring in extreme environments using a low-cost, low-power, and robust monitoring solution. However, that study also identified several areas requiring further development, including improving system stability under high contact resistance conditions, enhancing data reliability and remote data transfer capabilities, and enabling adaptive acquisition strategies through remote system control. These challenges highlight the need for continued development of A-ERT systems specifically designed for harsh permafrost environments.

This study presents a year-round A-ERT dataset acquired from a high-altitude site near the Kumtor gold mine in the Central Tien Shan, Kyrgyzstan, located at approximately 3550 m a.s.l. Although interest in permafrost monitoring in Central Asia has increased in recent years, the Tien Shan remains one of the least instrumented permafrost regions globally, with existing observations often limited to point measurements or short-term campaigns [4]. Introducing continuous and spatially distributed observations improves the understanding of permafrost dynamics and facilitates the validation of numerical models in the region. Building on previous system developments, this work evaluates the performance of an improved A-ERT system under harsh high-mountain conditions, with particular focus on system robustness, data quality, remote operation, and long-term autonomous monitoring. In addition, the study investigates the temporal evolution of subsurface resistivity and its relationship with temperature variations. By combining technological development with field-scale monitoring, this work provides both a technical assessment of A-ERT capabilities and new insights into subsurface freeze–thaw processes in a region where continuous geophysical observations remain scarce.

## 2. Study Area

**Figure 1:**
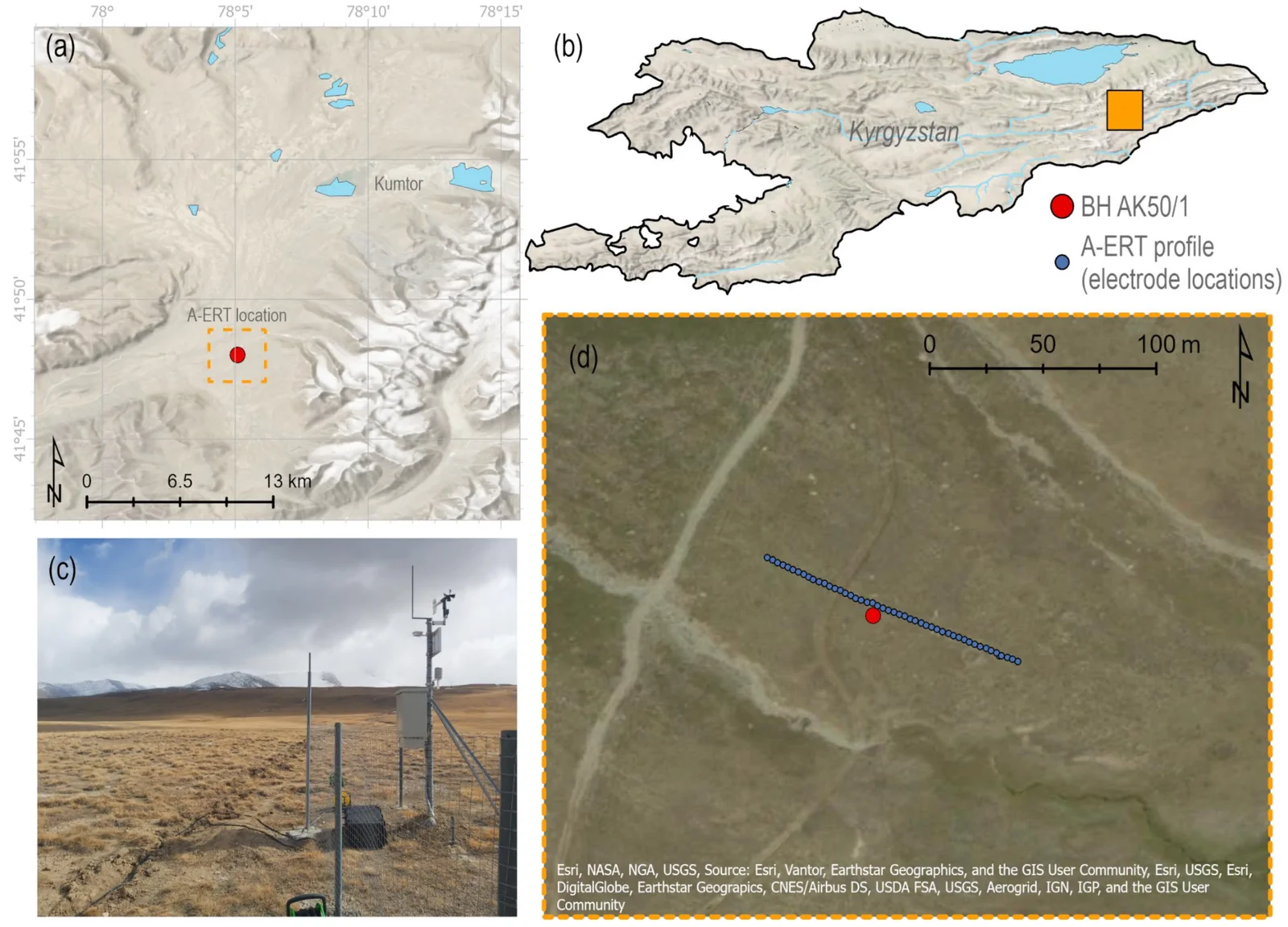
Study site and measurement setup in the Central Tien Shan, Kyrgyzstan. (**a**) Regional overview showing the location of the A-ERT monitoring site relative to the Kumtor Gold Mine. (**b**) Location of the study area within Kyrgyzstan. (**c**) Photograph of the A-ERT monitoring site, including the meteorological station. (**d**) Detailed view of the study site showing the A-ERT profile (blue dots representing electrode locations) and borehole BH AK50/1 (red circle).

The study site (Figure 1) is located on the Arabel plateau near the Kumtor gold mine in the Central Tien Shan, Kyrgyzstan, at an elevation of 3550 m a.s.l. The study site is characterized by a cold, semi-arid high-mountain climate and widespread mountain permafrost [4]. Mean annual air temperature is approximately −6.8 °C, while annual precipitation ranges between approximately 290 and 350 mm, with a substantial proportion falling as snow. The combination of high elevation, continental climatic conditions, and relatively low precipitation promotes the persistence of permafrost across large parts of the plateau environment. The A-ERT monitoring site is situated on a gentle slope composed predominantly of fine-grained sediments (silt and sand) with sparse vegetation cover. In contrast to coarse blocky or rockwall mountain permafrost environments frequently investigated using geophysical methods (e.g., [18,31]), the Arabel plateau is characterized by silty to fine-grained materials with relatively high moisture retention capacity and volumetric ground ice contents estimated between 20% and 50% in the upper permafrost [4]. The active-layer thickness at the site typically ranges between 1.5 and 1.8 m. The depth of zero annual amplitude (ZAA) is situated around 10–15 m depth with temperatures near −1.5 °C [17,32], with total permafrost thickness documented to exceed 100 m in the Kumtor region [2].

## 3. Materials and Methods

### 3.1. A-ERT System

**Figure 2:**
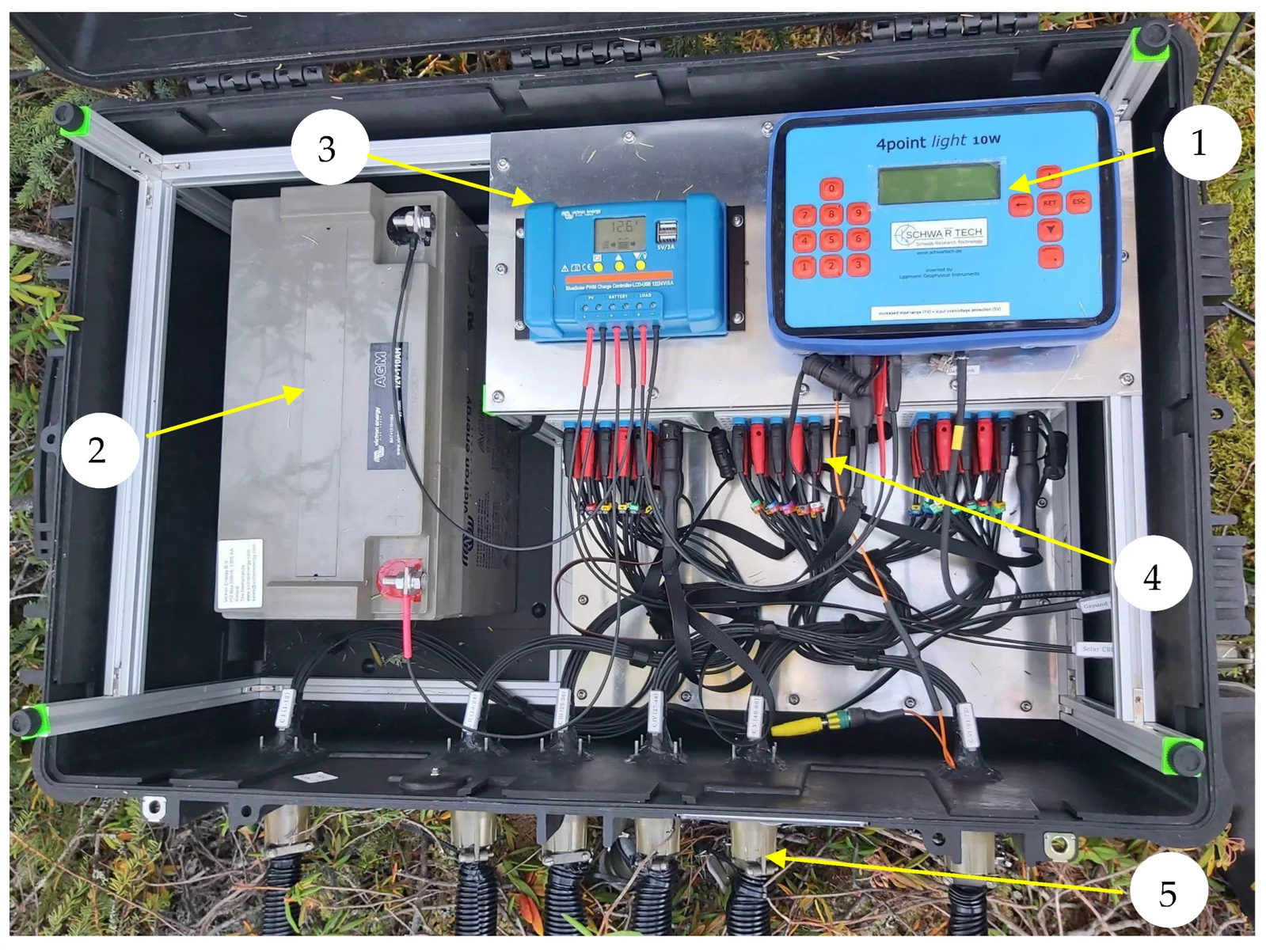
The latest version of the TRACE (Time-lapse Resistivity Assessment for Critical Environments) autonomous Electrical Resistivity Tomography (A-ERT) monitoring system. The system comprises a lightweight 4-point Light 10 W resistivity meter integrated with the remote communication module (1), a solar-powered battery (2) with charge controller (3), switch boxes for multi-electrode measurements (4), and military-grade connectors (5) housed within a customized IP67 and MIL-STD-810F weather-resistant enclosure designed for long-term autonomous operation in harsh permafrost environments.

The A-ERT system, referred to here as TRACE (Time-lapse Resistivity Assessment for Critical Environments), consists of a lightweight 4-point Light 10 W resistivity meter (Lippmann and Schwab Research Technology) and switch boxes enabling multi-electrode measurements, a solar-powered battery system, a two-way remote communication datalink (Schwab Research Technology), military-grade connectors, UV- and temperature-rated cables, and a customized weather-resistant enclosure (IP67- and MIL-STD-810F-rated) housing the main electronic components (Figure 2). The system was developed as a permanent monitoring installation for long-term autonomous operation under harsh high-mountain permafrost conditions.

TRACE is designed as a low-power monitoring system with an output current range between 1 μA and 100 mA. The minimum transmitter current of 1 μA is approximately one order of magnitude lower than the current resolution typically available in conventional commercial resistivity systems, making the system particularly suitable for monitoring the extremely high resistivities commonly encountered in frozen ground without overloading the receiver circuitry [29]. The number of switch boxes is flexible and can be adapted according to the number of electrodes required for a specific monitoring configuration.

TRACE builds upon earlier A-ERT prototype systems described in detail in Farzamian et al. [30], while incorporating several hardware and operational improvements developed to address limitations identified during previous deployments in Antarctica and other extreme environments [30]. Earlier field experiments demonstrated the feasibility of low-cost and low-power A-ERT monitoring, but also highlighted challenges associated with high contact resistance, signal saturation, limited remote accessibility, and reduced measurement stability under highly resistive frozen conditions [30]. These limitations became particularly critical during winter periods when ground resistivity increased substantially as conductive pathways were reduced due to pore-water freezing.

To improve system performance under such conditions, several modifications were implemented in the current TRACE configuration. To mitigate errors associated with inadequate reference electrode contact, which become pronounced during periods of high contact resistance, the acquisition electronics were redesigned. In particular, 100 MΩ input resistors were introduced into the switch boxes to reduce dependence on grounding electrodes and improve measurement stability during potential measurements. In addition, the voltage input range was expanded to ±1 V to enable stable acquisition under the elevated voltage conditions expected in highly resistive environments. These modifications were specifically aimed at reducing signal saturation and improving data quality during winter monitoring periods. Additional protective electronic components were also incorporated to improve system resilience against lightning-related electrical disturbances, which are expected in exposed high-mountain environments.

The latest TRACE configuration also incorporates an upgraded remote communication architecture enabling real-time data transfer, remote monitoring of system status, and dynamic modification of acquisition parameters without requiring field access. This includes the ability to remotely adjust acquisition settings such as electrode configuration (e.g., Wenner, Dipole–Dipole or Schlumberger arrays), measurement frequency, current injection limits, stacking parameters, and survey timing in response to environmental conditions or power availability. These capabilities substantially improve operational flexibility and reduce the logistical constraints typically associated with long-term monitoring in remote permafrost regions. One important application of this functionality is the ability to increase acquisition frequency during freeze–thaw transition periods when subsurface conditions evolve rapidly.

### 3.2. Data Acquisition

The monitoring profile at the Kumtor site consists of 48 permanently installed stainless steel electrodes distributed along a 94 m profile, with 2 m spacing, across fine-grained sediments. A Wenner electrode configuration was selected to reduce the number of measurements required for each monitoring cycle, thereby reducing energy consumption during autonomous operation, while providing a relatively high signal-to-noise ratio under the highly resistive conditions encountered in frozen ground [33]. The Wenner array also provides good sensitivity to vertical variations in subsurface resistivity beneath the centre of the array [34], which is advantageous for resolving the depth-dependent changes associated with the active layer and underlying permafrost. These characteristics made the Wenner configuration a practical compromise between measurement efficiency, signal quality, and sensitivity to the targeted depth range for long-term autonomous monitoring.

The permanent installation enabled repeated measurements using identical electrode geometry throughout the monitoring period, thereby minimizing geometric uncertainties between successive datasets and improving the consistency of time-lapse analysis. The system was configured for fully autonomous operation, allowing continuous acquisition of resistivity measurements throughout the seasonal freeze–thaw cycle.

The system was installed in October 2023 without the remote communication module, and data from the first monitoring campaign were downloaded manually during a subsequent site visit in late August 2024. In the present study, only data acquired during the first monitoring campaign (October 2023 to August 2024) are presented, providing an almost complete annual record for investigating seasonal active-layer and permafrost dynamics.

Ground temperature and meteorological data were obtained from the existing permafrost monitoring infrastructure installed at the site prior to the installation of the A-ERT system. Air temperature was measured at an automatic weather station, while ground temperatures were recorded in a borehole instrumented with temperature sensors at multiple depths down to 30 m [17,32]. Both installations were located in the vicinity of the A-ERT profile, approximately 3–5 m from its midpoint. For the analyses presented in this study, temperatures at 0.5, 1, 2, 3, 5, and 10 m depth were used to investigate the relationship between subsurface thermal conditions and resistivity variations derived from the A-ERT monitoring system. All temperature and meteorological datasets were quality-controlled prior to analysis.

### 3.3. A-ERT Data Processing and Inversion

Due to the high temporal resolution of the autonomous monitoring setup and the large number of datasets generated during continuous operation, a semi-automated processing workflow was implemented following the general methodology proposed by Herring et al. [8] and further developed for A-ERT applications by Farzamian et al. [25]. The workflow was designed to efficiently process large time-series resistivity datasets while ensuring consistent quality control and minimizing subjective manual intervention. Such automated procedures are particularly important for long-term A-ERT deployments, where the number of repeated measurements can reach several hundreds or thousands of datasets per year, making manual inspection impractical.

The automated filtering workflow consisted of several sequential quality-control steps adapted from the workflow presented by Farzamian et al. [25]. Measurements with negative apparent resistivities, stacking errors exceeding 10%, or extreme apparent resistivity values (>9 standard deviations from the dataset distribution) were removed. A moving-median filter was then applied in log-resistivity space, rejecting measurements deviating by more than 7% from the local median response. Electrodes associated with more than 30% rejected measurements were classified as faulty, and all measurements involving those electrodes were excluded. Compared to filtering approaches based solely on stacking error, the moving median filter is an effective additional step for identifying anomalous measurements [35]. This is because unrealistic spatial behavior can still be associated with stable stacking statistics [36]. Finally, datasets with an excessive proportion of removed measurements (>30%) were excluded entirely from inversion, as highly incomplete datasets may introduce instability and artifacts in time-lapse inversion results. Similar approaches have been shown to substantially improve inversion stability and consistency for automated permafrost monitoring datasets (e.g., [25]).

### 3.4. A-ERT Data Inversion

Following filtering, all datasets were inverted using the open-source pyGIMLi framework [37], incorporating the topography data [38]. Similarly to Farzamian et al. [25], an L1-norm (“blocky”) regularization scheme [39] was selected in order to better preserve sharp resistivity contrasts associated with the transition between thawed active-layer material and underlying frozen ground. Compared to smoothness-constrained inversion approaches, blocky regularization is generally more suitable for permafrost applications where abrupt boundaries with high resistivity contrast, i.e., between frozen and unfrozen zones, may exist [25].

The inversion procedure employed a cascaded time-lapse approach, whereby each inverted model served as the starting model for the subsequent time step. This strategy improves temporal consistency between consecutive inversions and reduces inversion instability for large time-series datasets [40,41]. The initial model for the first dataset was defined as a homogeneous half-space using the average apparent resistivity of the corresponding survey. Data errors were estimated using a linear noise model consisting of a 4% relative error component and a small absolute error term (0.001 Ωm), values that are commonly adopted in ERT studies where detailed reciprocal-error analyses are unavailable (e.g., [8]). The inversion process was iterated until convergence criteria were satisfied, including acceptable data misfit levels or minimal changes in the objective function between iterations.

In addition to the analysis of apparent and inverted resistivity distributions, several complementary analyses were performed to investigate the relationship between electrical resistivity and subsurface thermal conditions. Resistivity evolution was examined both spatially and temporally through virtual borehole extraction, depth-dependent time-series analysis, and averaging within selected zones of interest. Virtual borehole analysis enabled direct comparison between inverted resistivity evolution and measured ground temperatures, facilitating assessment of freeze–thaw propagation with depth (e.g., [25]).

## 4. Results and Discussion

### 4.1. System Performance and Data Quality Assessment

**Figure 3:**
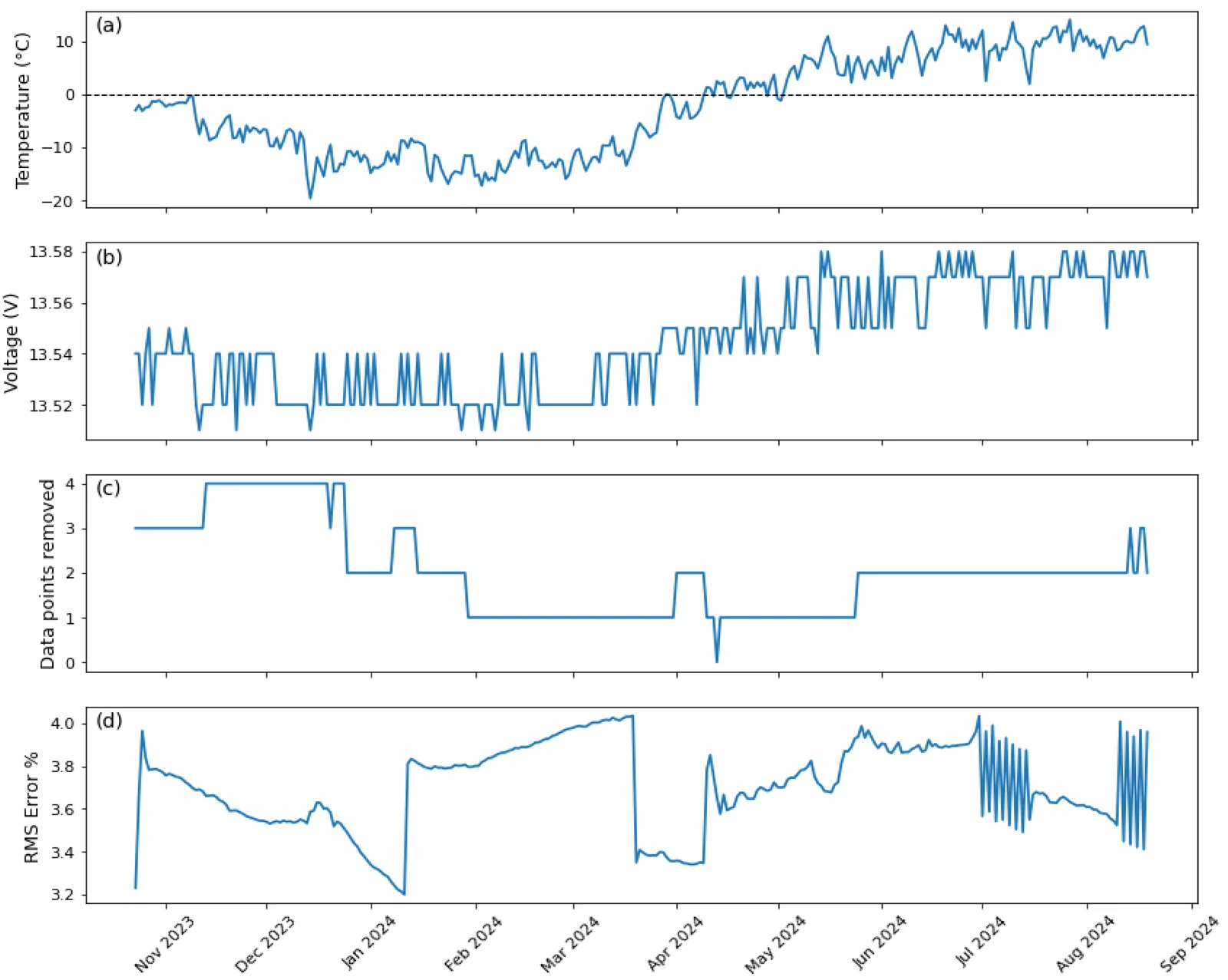
Time series from October 2023 to August 2024 showing: (**a**) internal temperature recorded by the 4-point light 10 W resistivity meter within the TRACE setup; (**b**) battery voltage evolution during autonomous operation; and data-quality indicators including (**c**) the number of filtered data points removed from each dataset and (**d**) the RMS error of the inverted A-ERT models. The horizontal dashed line in (**a**) indicates the 0 °C threshold.

Figure 3 summarizes the operational performance of the TRACE system and the quality of the datasets acquired between October 2023 and August 2024, including the internal temperature of the resistivity meter, battery voltage, the number of filtered data points removed during processing, and the root-mean-square (RMS) error of the inverted models.

**Figure 4:**
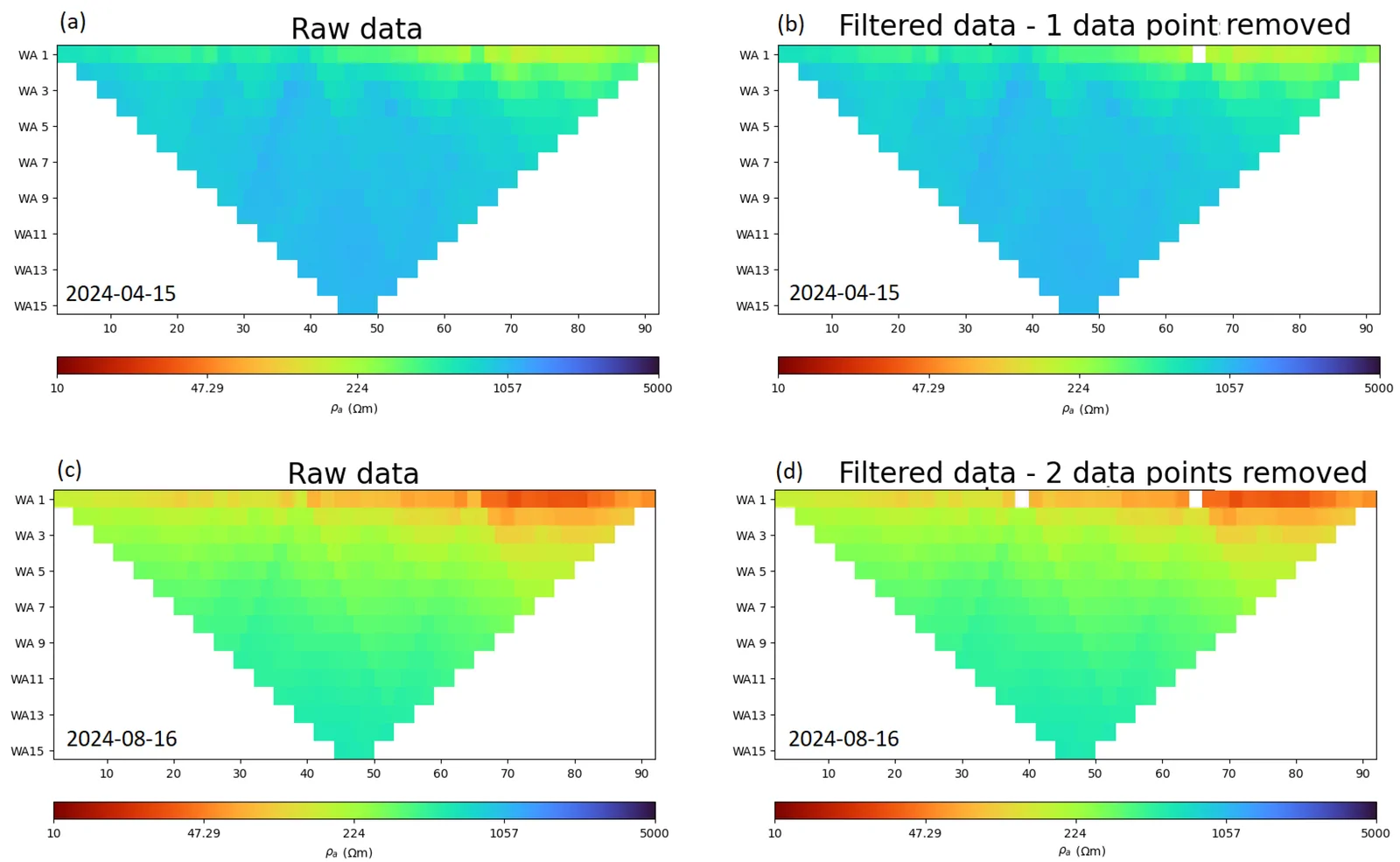
Representative pre-inversion apparent resistivity (ρ_a_) pseudosections under cold and warm ground-temperature conditions: raw and filtered data for (**a**,**b**) 15 April 2024, corresponding to the coldest ground-temperature conditions during the monitoring period, and (**c**,**d**) 16 August 2024, corresponding to the warmest ground-temperature conditions. The raw and filtered datasets are shown before and after quality-control filtering, respectively.

The internal temperature recorded within the system enclosure (Figure 3a) demonstrates that the monitoring system operated continuously under extreme seasonal conditions characteristic of the high-altitude permafrost environment. External air temperatures dropped below −30 °C during winter (cf. Figure 4a), while internal enclosure temperatures remained above −20 °C. The observed temperature offset likely reflects a combination of factors, including the insulating properties of the weather-resistant enclosure, heat dissipation from the electronic components, seasonal snow insulation during winter, and solar heating of the dark-colored housing during periods of high incoming radiation. Although internal temperatures remained below freezing for extended periods during winter, the system continued autonomous acquisition without operational interruption, indicating robust performance of both the electronic components and power-management system under prolonged sub-zero conditions.

Battery voltage evolution (Figure 3b) indicates stable long-term power supply throughout the monitoring period. Despite harsh winter conditions and reduced solar radiation during winter months, the solar-powered battery system was fully charged to support continuous autonomous operation. Battery voltage showed no large fluctuation thanks to the solar panel regulator and no evidence of power failure or critical discharge events were recorded. These results demonstrate the suitability of the low-power TRACE architecture for long-term deployment in remote environments with limited site accessibility.

The applied processing workflow resulted in only a small proportion of measurements being excluded from the final datasets (Figure 3c). Out of 360 apparent resistivity (ρ_a_) measurements acquired per dataset, typically only 1–4 measurements were removed during filtering, corresponding to approximately 1% or less of the total acquired data. The low percentage of filtered measurements indicates a high overall data quality and generally low contact resistances throughout the year despite the challenging environmental conditions in frozen ground environments.

To further illustrate the quality of the ρ_a_ measurements prior to inversion, Figure 4 presents representative raw and filtered apparent resistivity pseudosections for the coldest and warmest ground-temperature conditions observed during the monitoring period (15 April and 16 August 2024, respectively). The raw pseudosections show coherent spatial patterns across the profile, with only a small number of measurements subsequently removed during quality control. Comparison of the raw and filtered datasets demonstrates that filtering had a limited effect on the overall apparent resistivity structure, with only one measurement removed from the April dataset and two from the August dataset. These observations further support the high consistency of the acquired data under both cold and relatively warm ground conditions.

The RMS error of the inverted datasets remained relatively stable throughout the monitoring period, generally ranging between 3% and 4% (Figure 3d). These values indicate very good agreement between observed and modeled apparent resistivity data and confirm a good overall data quality. Slight seasonal variations in RMS error are likely associated with the higher resistivities and reduced signal-to-noise ratios characteristic of frozen conditions, as well as enhanced temporal variability during freeze–thaw transitions. Jumps in RMS errors are likely a result of slight variations in the inversion process, where some datasets may reach convergence one iteration later or earlier than the previous time step, causing differences in RMS error values.

### 4.2. Seasonal Evolution of Apparent Resistivity and Temperature

**Figure 5:**
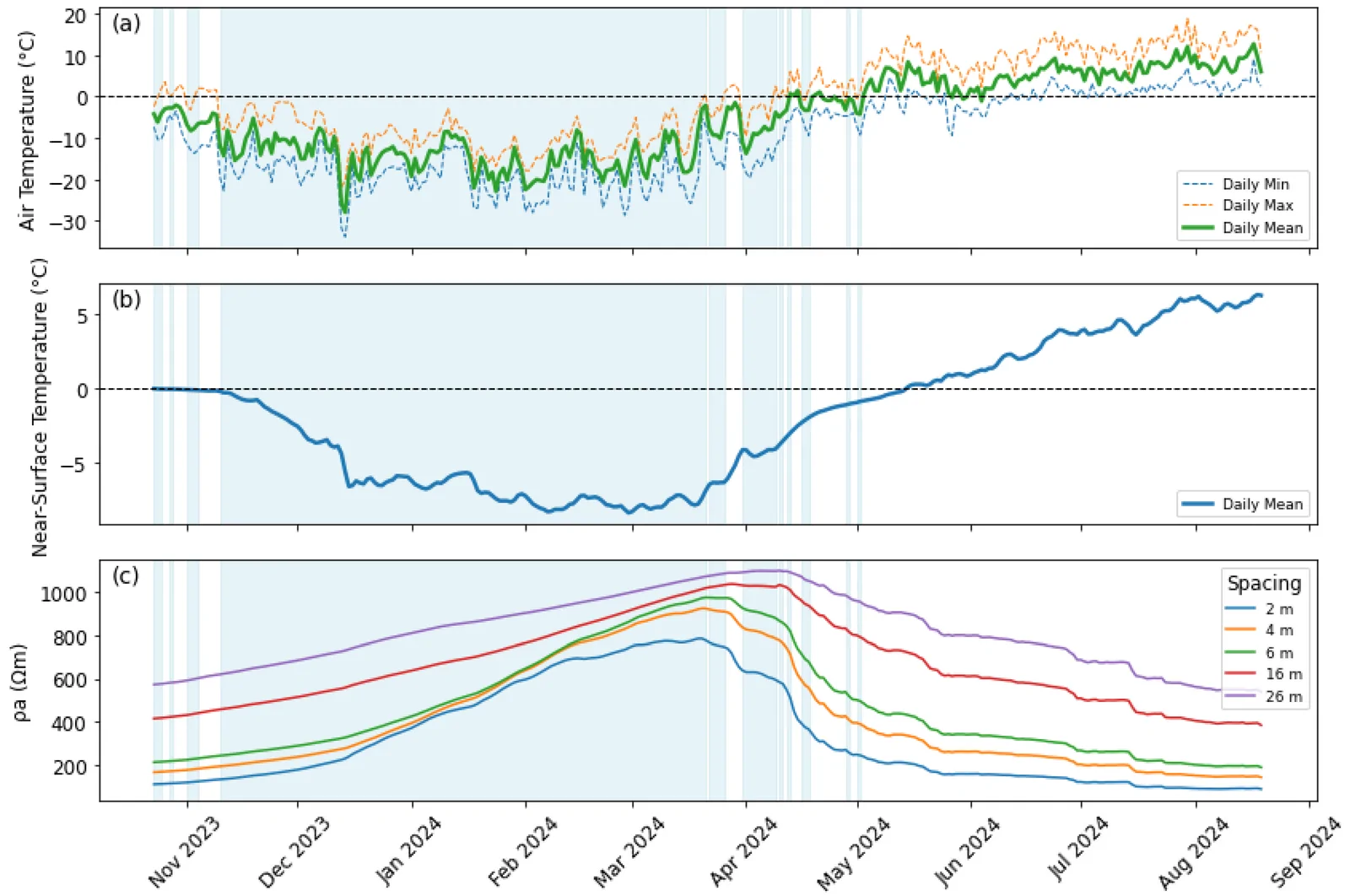
Time series from October 2023 to August 2024 showing: (**a**) air temperature; (**b**) near-surface ground temperature at 0.5 m depth; and (**c**) average apparent resistivity (ρ_a_) at multiple electrode spacings. Blue shading indicates periods with daily maximum air temperature below 0 °C. The horizontal dashed line in (**a**) indicates the 0 °C threshold.

Figure 5 presents the temporal evolution of air temperature (daily minimum, mean, and maximum), near-surface ground temperature at 0.5 m depth, and average ρ_a_ for selected quadrupoles between October 2023 and August 2024. The figure also highlights periods with negative maximum daily air temperatures and illustrates the seasonal evolution of near-surface thermal and electrical conditions at the site.

Air temperature exhibited large variability during the monitoring period, ranging from −33.9 to 18.9 °C. Near-surface ground temperature showed damped variations compared to atmospheric conditions, ranging from −8.5 to 6.3 °C, with negligible daily fluctuations.

Average ρ_a_ displayed a pronounced seasonal cycle across all investigated electrode spacings (Figure 5c). In general, resistivity increased progressively during autumn and winter as ground temperatures decreased and conductive pore-water pathways became increasingly restricted by freezing. Maximum resistivity values were observed during late winter and early spring, followed by a rapid decrease during thawing conditions in spring and early summer. This overall pattern demonstrates the strong sensitivity of electrical resistivity to seasonal freeze–thaw dynamics.

The magnitude and variability of resistivity differed substantially with electrode spacing. The shallowest measurements, corresponding to the 2 m electrode spacing (~1 m depth of investigation), exhibited the largest temporal variability, with apparent resistivity values ranging from 86 to 785 Ωm. In contrast, larger electrode spacings, which sample deeper subsurface regions, showed progressively higher mean resistivity values with reduced seasonal variability, demonstrating that shallow subsurface layers are more strongly influenced by seasonal freeze–thaw processes, whereas deeper permafrost layers remain thermally buffered and comparatively stable throughout the year. Shallow apparent resistivity values reached maximum levels during mid- to late March, whereas deeper spacings continued increasing until April, reflecting the expected delayed propagation of freezing conditions with depth.

During the sustained freezing period in late October 2023, near-surface ground temperatures remained close to the thawing point for an extended period while shallow resistivity constantly increased, indicating the presence of zero-curtain conditions associated with progressive freezing of pore ice.

Several short-term resistivity fluctuations were observed during transitional periods, particularly in spring. Temporary decreases in shallow apparent resistivity coincided with short-lived warming events with maximum daily air temperatures near or >0 °C, despite near-surface ground temperatures remaining predominantly below 0 °C (e.g., during late March 2024). These observations suggest that electrical resistivity is highly sensitive to transient changes in unfrozen water content and moisture connectivity within the active layer. These findings are also in agreement with other studies in different environments of Antarctica (e.g., [25,30]), demonstrating the potential of A-ERT in studying the impact of short-lived meteorological events on active layer and permafrost dynamics.

### 4.3. Seasonal Evolution of 2D Inverted Resistivity Models

**Figure 6:**
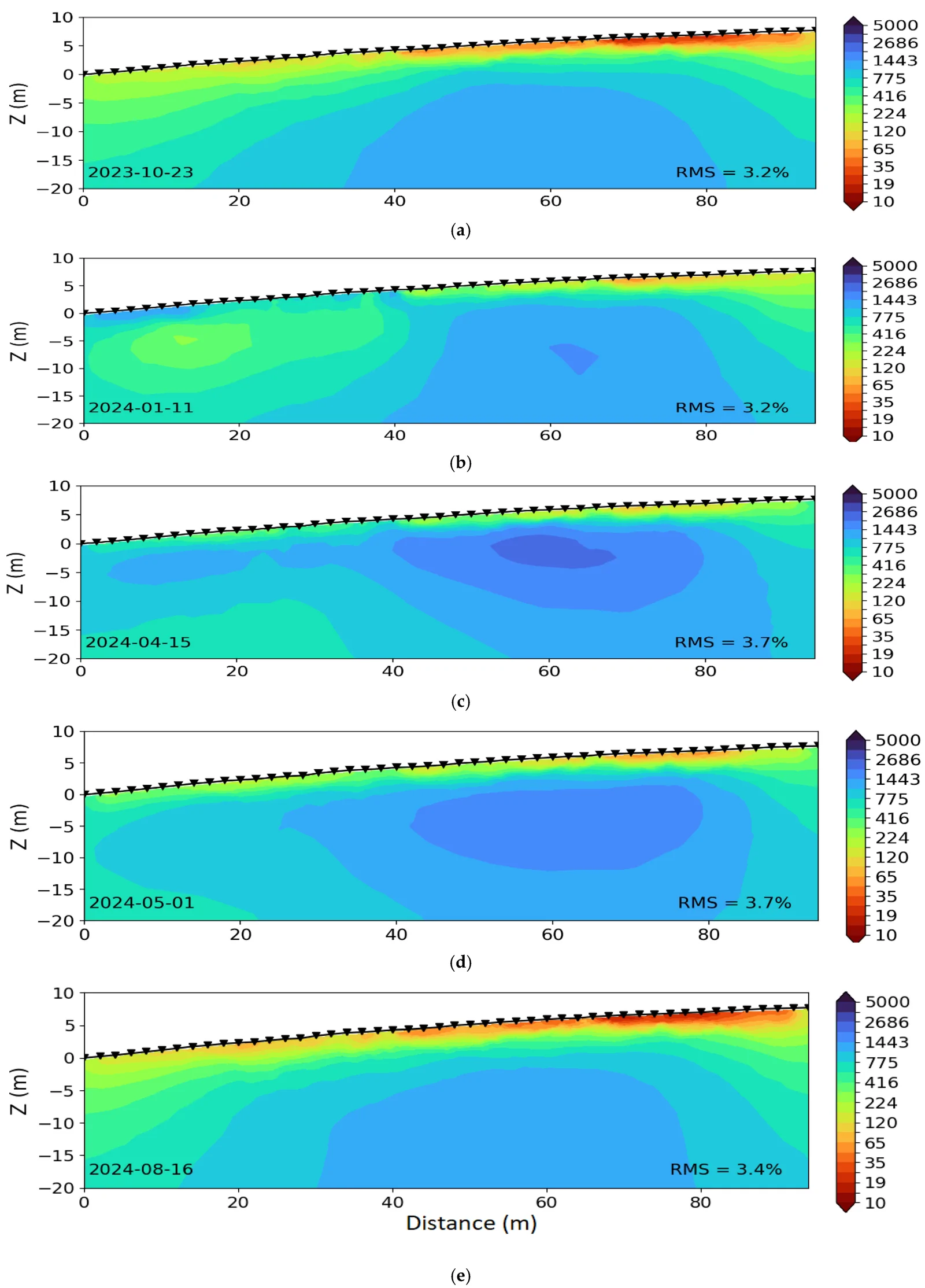
Selected inverted 2D resistivity models illustrating the seasonal evolution of subsurface electrical resistivity between October 2023 and August 2024: (**a**) late October 2023, representing early freezing conditions; (**b**) mid-January 2024, corresponding to winter conditions; (**c**) mid-April 2024, during late winter to early spring transition; (**d**) early May 2024, representing the onset of thawing conditions; and (**e**) mid-August 2024, corresponding to late summer and maximum thaw conditions.

Selected inverted 2D resistivity models illustrating the seasonal evolution of subsurface electrical resistivity between October 2023 and August 2024 are presented in Figure 6. The selected models represent key stages of the annual freeze–thaw cycle and provide a spatial overview of temporal resistivity variations within the active layer and underlying permafrost.

Figure 6a shows the first A-ERT survey acquired in late October 2023 and serves as the reference model at the beginning of the monitoring period. At this stage, near-surface freezing had already started, although shallow resistivity values remained comparatively low. Figure 6b shows the model from mid-January 2024, representing winter conditions when ground temperatures within the active layer had decreased substantially below 0 °C and apparent resistivity values began increasing in the shallow subsurface. The third model (Figure 6c), corresponding to mid-April 2024, represents the late winter to early spring transition period during which the highest resistivity values were observed, particularly within deeper portions of the profile. This timing corresponds well with the maximum apparent resistivity values observed in the temporal analysis (Figure 5c). In contrast, the fourth and fifth examples (Figure 6d,e), representing early May 2024 and mid-August 2024, illustrate the onset of thawing conditions and late summer conditions, respectively. The early May model coincides with the zero-curtain period and resistivity decrease within the active layer, while the August model reflects maximum seasonal thaw depth and minimum shallow resistivity values.

The inverted models reveal a relatively conductive near-surface zone within approximately the upper 2 m of the subsurface, consistent with the maximum active-layer thickness of 1.8 m reported for the site [4]. This shallow zone exhibits large temporal variability throughout the monitoring period, indicating strong sensitivity to seasonal freeze–thaw dynamics and associated changes in liquid water content, as also observed in shallow ρa data (cf. Figure 5c). Below the active layer, corresponding to the underlying permafrost, resistivity variations become progressively smaller with depth, reflecting the attenuation of seasonal thermal fluctuations. Nevertheless, clear seasonal evolution remains visible within the upper permafrost zone, particularly between approximately 2 and 10 m depth. Resistivity increases during winter and early spring indicate continued cooling and gradual reduction in unfrozen water content even under permanently frozen conditions. At greater depths below approximately 10 m, resistivity variability becomes comparatively limited, suggesting thermally stable permafrost conditions with minimal seasonal influence.

One notable feature of the models is the conductive behavior of the shallow subsurface during winter conditions. Compared with the coarse-grained permafrost environments, where freezing often produces very sharp resistivity increases in the active layer (e.g., [25,30]), the active layer at the Kumtor site remains comparatively conductive throughout much of the winter period. This behavior likely reflects both the fine-grained nature of the sediments and the occurrence of short-lived warming events during winter and spring, as seen in Figure 5. Fine-grained soils are capable of retaining substantial amounts of unfrozen water below 0 °C due to capillary and adsorptive effects, thereby maintaining electrically conductive pathways even under frozen conditions [42]. As a result, resistivity evolution appears more gradual and less abrupt than typically observed in, e.g., the more coarse-grained substrates in the European Alps (cf. [18,24]).

The models also reveal lateral variability along the profile, with stronger temporal dynamics observed particularly in the left portion of the section. This spatial heterogeneity likely reflects local variations in soil properties, or moisture distribution, and variations in ground ice content, which was also indicated by refraction seismic data and ice content modelling in Mathys et al. [4].

### 4.4. Depth-Dependent Temperature–Resistivity Relationships and Hysteresis

**Figure 7:**
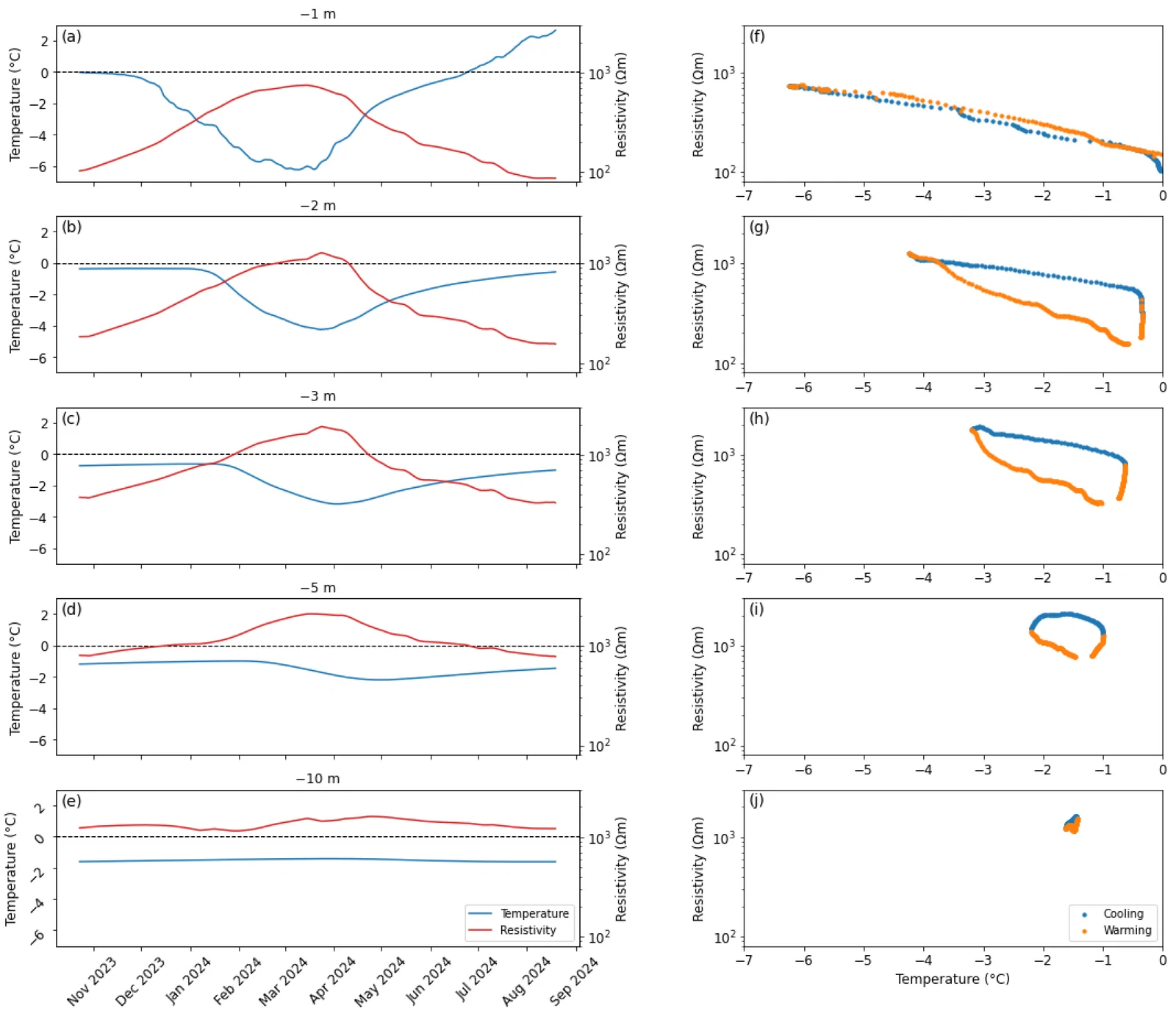
Temporal evolution of ground temperature (blue) and extracted resistivity values (red) (left column) together with the corresponding temperature–resistivity relationships during cooling and warming periods (right column) at selected depths between October 2023 and August 2024. Panels (**a**–**e**) show the time series at depths of 1, 2, 3, 5, and 10 m, respectively, while panels (**f**–**j**) present the corresponding temperature–resistivity relationships. Dashed horizontal lines in panels (**a**–**e**) indicate the 0 °C threshold. Only sub-zero temperature data are shown in the temperature–resistivity relationships (**f**) for the 1 m depth.

Figure 7 presents the temporal evolution of ground temperature and extracted resistivity values at the virtual borehole (located at the midpoint of the A-ERT profile, close to the borehole) together with the corresponding temperature–resistivity relationships during cooling and warming periods. The combined analysis reveals clear depth-dependent differences in the thermal–electrical response of the subsurface and demonstrates the progressive development of hysteresis below the active layer.

At 1 m depth (Figure 7a,f), corresponding to the active layer, temperature exhibited the largest seasonal variability, ranging from −6.3 to 2.7 °C with a standard deviation of 2.6 °C. Resistivity varied between approximately 86 and 744 Ωm (standard deviation 221 Ωm) and reached its maximum values (>700 Ωm) during mid-March, coinciding with the minimum ground temperatures (<−6 °C). The resistivity evolution closely followed the seasonal thermal cycle, increasing during autumn and winter as temperatures decreased and declining rapidly during spring thawing. The relatively synchronous behavior of temperature and resistivity, together with the limited separation between the cooling and warming trajectories, indicates that electrical properties at this depth are primarily controlled by seasonal freezing and thawing processes affecting liquid water content and pore connectivity within the active layer.

One notable feature is the apparent autumn zero-curtain period observed between late October and mid-November 2023, during which ground temperature remained close to the freezing point while resistivity continued to increase gradually, suggesting that phase-change processes were still ongoing despite limited temperature variations. However, the onset of freezing was not fully captured because monitoring began in late October 2023, after part of the autumn freeze-up period had already occurred. In contrast, only a weak zero-curtain signature was observed during spring thawing, when both temperature and resistivity transitioned relatively rapidly from winter to summer conditions. This behavior differs from observations at Antarctic monitoring sites reported by Farzamian et al. [25,30], where the thawing-season zero-curtain was more prolonged than the freezing-season zero-curtain. Although snow-cover observations are not available for the Kumtor site, differences in snow accumulation and its insulating effect may partly explain these contrasting seasonal responses.

At 2 and 3 m depth (Figure 7b,c,g,h), temperatures remained entirely below 0 °C throughout the monitoring period, ranging from approximately −4.2 to −0.3 °C at 2 m and from −3.2 to −0.6 °C at 3 m. Despite these comparatively small thermal variations, resistivity exhibited pronounced seasonal changes, reaching maximum values of approximately 1280 Ωm and 1910 Ωm, respectively, with standard deviations exceeding 340 Ωm and 460 Ωm. Although the timing of maximum resistivity generally coincided with the lowest ground temperatures, the seasonal response became progressively delayed relative to the active layer. The corresponding temperature–resistivity plots reveal a clear separation between cooling and warming trajectories, demonstrating that, for the same temperature, resistivity depends on the thermal history of the ground. This behavior suggests that below the active layer, electrical properties are no longer controlled solely by temperature, but are also influenced by gradual changes in unfrozen water content, pore-scale connectivity, and ice distribution within the fine-grained permafrost.

At 5 m depth (Figure 7d,i), temperature variability was strongly attenuated, remaining between approximately −2.2 and −1.0 °C (standard deviation 0.42 °C), whereas resistivity continued to vary substantially between approximately 780 and 2100 Ωm (standard deviation > 400 Ωm). Furthermore, the resistivity maximum occurred earlier than the temperature minimum, indicating an increasing decoupling between thermal and electrical responses. The hysteresis loop also became more pronounced despite the relatively narrow temperature range, suggesting that seasonal changes in unfrozen water distribution and pore connectivity continue to influence the electrical properties of the permafrost.

At 10 m depth (Figure 7e,j), temperature remained nearly constant throughout the monitoring period, varying only between −1.62 and −1.43 °C with a standard deviation of 0.07 °C. Resistivity variability was also substantially reduced compared with shallower layers, and only minor hysteresis was observed. These results indicate a thermally stable permafrost zone with limited seasonal influence.

Overall, the results demonstrate a progressive transition from direct thermal control of resistivity within the active layer to increasingly complex thermo-hydrological controls within the underlying permafrost. The observed hysteresis is consistent with previous studies of fine-grained permafrost. For example, Tomaškovičová and Ingeman-Nielsen [43] reported that, for the same sub-zero temperature, resistivity during freezing is generally higher than during thawing because ice formation progressively disconnects conductive pore-water pathways, whereas thawing reconnects these pathways before complete melting occurs. Consequently, the relationship between resistivity and temperature is not unique but depends on the thermal history of the ground. Similar processes may explain the hysteresis observed at the Kumtor site, although its magnitude appears less pronounced than that reported for Greenland permafrost. These findings further suggest that the use of a single petrophysical relationship to estimate unfrozen water content from resistivity measurements may introduce systematic errors and that separate calibrations for freezing and thawing conditions may be required [44].

### 4.5. A-ERT-Related Uncertainties and Operational Limitations

Several methodological and operational factors should be considered when interpreting the A-ERT results obtained at the Kumtor site. First, the spatial resolution and depth sensitivity of the monitoring configuration are inherently limited by the 2 m minimum electrode spacing. The minimum electrode spacing limits sensitivity to very shallow near-surface processes, and freezing within the uppermost decimeters may therefore be only partially resolved in the inverted models. In addition, ERT sensitivity generally decreases with increasing depth and towards the ends of the profile, resulting in greater uncertainty in these regions. Consequently, resistivity variations in the deeper parts of the model, as well as those close to the profile boundaries, should be interpreted with greater caution.

These limitations are particularly relevant at the Kumtor site because of the strong resistivity contrast between the relatively conductive active layer and the resistive underlying permafrost. Such contrasts can reduce the sensitivity of the inversion to deeper regions and increase the dependence of the recovered resistivity structure on the better-constrained overlying layers. Consequently, part of the magnitude of the resistivity variations observed at depth, including the pronounced hysteresis between cooling and warming periods, may be influenced by inversion sensitivity and model trade-offs. This is particularly relevant at 5 and 10 m depth, where relatively small temperature variations are associated with comparatively large changes in inverted resistivity. The magnitude of these deep resistivity variations should therefore not be interpreted quantitatively without considering the associated inversion uncertainty.

Additional uncertainty arises from the fact that electrical resistivity is affected not only by temperature and unfrozen water content but also by sediment composition, clay content, mineralogy, and pore-water ionic concentration. These factors may contribute to the lateral resistivity variability observed along the profile, including the relatively conductive zone in the shallow subsurface. Detailed spatially resolved information on sediment properties and pore-water chemistry was not available for the monitoring profile, and their individual contributions to the observed resistivity response therefore cannot be separated. Site-specific laboratory measurements and petrophysical investigations would be valuable for quantifying these relationships and for distinguishing physical hysteresis from inversion-related effects.

From an operational perspective, the TRACE system performed reliably throughout the monitoring period, with continuous acquisition and less than 1% of measurements removed during quality control. The main challenges associated with long-term A-ERT monitoring in such environments are maintaining the robustness and functionality of the system under harsh environmental conditions, ensuring continuous power supply, particularly during winter when solar energy availability is limited, and maintaining measurement stability under high electrode contact resistance and elevated subsurface resistivity. The latter conditions can require operation at very low injection currents. The improvements and adaptations to the system described by Farzamian et al. [30], together with the hardware modifications implemented in the present TRACE configuration, including the high-impedance input configuration, expanded voltage input range, and improved remote communication architecture, were specifically designed to address these challenges. Nevertheless, system performance remains dependent on electrode–ground contact conditions, subsurface resistivity, power availability, and local environmental conditions. These factors should therefore be considered when deploying A-ERT systems at other remote permafrost sites, particularly where substantially higher resistivities, poorer electrode contact, limited power availability, or more severe environmental conditions are expected.

The power budget also provides scope for further optimization of the monitoring strategy. Throughout the monitoring period, the available battery capacity remained sufficient to support the implemented acquisition schedule, indicating that the system operated within its available energy budget. This suggests that, depending on site conditions and power availability, future deployments could increase the frequency of measurements, employ alternative electrode configurations, or incorporate additional measurements such as reciprocal measurements to improve data quality and monitoring robustness. Shorter acquisition intervals could also be considered during periods of rapid freeze–thaw transitions, when higher temporal resolution may provide additional information on transient subsurface processes.

## 5. Conclusions

This study presented the development and field validation of the TRACE A-ERT system for continuous subsurface monitoring under harsh high-mountain permafrost conditions in the Central Tien Shan, Kyrgyzstan. Throughout the monitoring period, the system maintained stable autonomous operation with no interruptions or operational failures. The results demonstrate that the system is capable of reliable long-term autonomous operation in remote environments characterized by extreme temperatures, limited accessibility, and strong seasonal freeze–thaw variability.

The acquired datasets revealed clear seasonal evolution of subsurface resistivity associated with freeze–thaw dynamics within the active layer and upper permafrost. Shallow subsurface resistivity exhibited strong seasonal variability and responded rapidly to atmospheric forcing, whereas deeper layers showed delayed and smoother responses reflecting attenuation of thermal signals with depth.

An important outcome of this study is the observation that resistivity evolution within the permafrost layer was not controlled solely by temperature. Despite permanently frozen conditions below the active layer, significant seasonal resistivity variations and pronounced temperature–resistivity hysteresis behavior were observed. These findings suggest that unfrozen water dynamics, pore-scale connectivity, and freeze–thaw history exert a strong influence on subsurface electrical properties within the fine-grained sediments of the Kumtor site.

Beyond the specific permafrost application presented here, the TRACE system demonstrates broader potential for long-term autonomous geophysical monitoring in remote and environmentally challenging settings. The combination of low-power operation, remote communication capabilities, adaptive acquisition control, and robust field performance makes the system suitable for a wide range of environmental, hydrological, cryospheric, and infrastructure monitoring applications where continuous subsurface observations are required. In parallel, the future development of a web-based platform for processing and inversion of A-ERT data may enable near real-time subsurface imaging and interpretation. Such an integrated monitoring framework opens possibilities for autonomous early-warning systems, continuous assessment of subsurface dynamics, and scalable deployment of geophysical observatories in remote environments.

## Data Availability

The A-ERT data can be found at Farzamian 2026 [45].
